# Predicting the Implications of Climatic Alterations on the Distribution of Endangered Species: A Case Study of Saxifragaceae on the Qinghai‐Xizang Plateau

**DOI:** 10.1002/ece3.71899

**Published:** 2025-08-06

**Authors:** Yang Lv, Zhaxi Cairang, Chenglin Sun, Xu Su, Yuping Liu, Yonghui Zhou, Kaiyue Wei, Xu Feng, Jieqiong Lei, Yinghui Zheng, Xuanlin Gao, Mir Muhammad Nizamani

**Affiliations:** ^1^ School of Life Sciences Qinghai Normal University Xining China; ^2^ Academy of Plateau Science and Sustainability Qinghai Normal University Xining China; ^3^ Key Laboratory of Biodiversity Formation Mechanism and Comprehensive Utilization of the Qinghai‐Xizang Plateau in Qinghai Province Qinghai Normal University Xining China; ^4^ Ganzhou Research Institute of Vegetables and Flowers Ganzhou China; ^5^ Institute of Marine Sciences, Guangdong Provincial Key Laboratory of Marine Disaster Prediction and Prevention Shantou University Shantou China

**Keywords:** climate change, climatic scenario, maximum entropy model, potential distribution, Saxifragaceae, shared socioeconomic pathway

## Abstract

Understanding the potential impacts of climate change on species distribution is crucial for the conservation of threatened taxa. The Saxifragaceae family, known for its susceptibility to habitat disturbance, exhibits a diverse distribution across multiple regions. While a significant proportion of this family is found on the Qinghai‐Xizang Plateau (QXP), nearly half of the Saxifraga species are native to Europe, with other genera, such as *Heuchera*, showing centers of diversity in North America and Japan. This study applies the Maximum Entropy (MaxEnt) model in combination with Shared Socioeconomic Pathways (SSPs) to assess the potential influence of climate change on the distribution and species richness of four endangered Saxifragaceae species ( *Saxifraga cernua* L., *Saxifraga tangutica* Engl., *Saxifraga przewalskii* Engl. ex‐Maxim., *Saxifraga unguiculata* Engl.) on the QXP, spanning from the Last Glacial Maximum to 2080. Our findings reveal that key environmental factors, including elevation, slope, mean annual temperature, isothermality, precipitation seasonality, and precipitation during the wettest quarter, significantly influence species distribution patterns. Historical climate models suggest that approximately 30% of the QXP provided highly suitable habitat for Saxifragaceae species, a proportion that has increased to over 30% in current projections, with this trend expected to persist across the next three time intervals. Optimal habitats were identified in the southeastern QXP, western Sichuan, and northern Yunnan. Projections indicate that these taxa will likely shift southward in response to ongoing climate changes. These results highlight the need for targeted conservation strategies, emphasizing the establishment of protected areas in southeastern QXP to preserve these vulnerable species of Saxifragaceae.

## Graphical abstract

In this study, we employ the Maximum Entropy (MaxEnt) model in conjunction with Shared Socioeconomic Pathways (SSPs) to assess the MaxEnt potential influence of climate change on the distribution and richness of four endangered Saxifragaceae species ( *Saxifraga cernua* L., Saxifraga tangutica Engl., Saxifraga przewalskii Engl. ex‐Maxim., Saxifraga unguiculata Engl.) on the QXP.

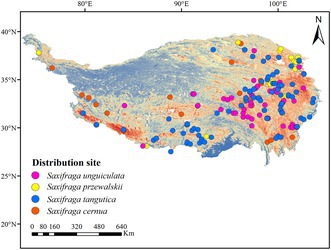

## 1 Introduction

The potential impacts of climate change on biodiversity and ecosystem functioning are of paramount concern, particularly as the world anticipates increasingly significant shifts in temperature and weather patterns (Bellard et al. 2012; Pecl et al. 2017). Evidence suggests that climate change is likely to alter plant distributions, with species expanding into newly suitable areas while declining in regions that become less hospitable (Kelly and Goulden 2008). Of particular concern is the shifting distribution of species within ecosystems already vulnerable to environmental change (Hooper et al. 2012). The Qinghai‐Xizang Plateau (QXP) serves as a prominent example of such a fragile habitat. Characterized by high altitudes, low oxygen levels, complex topography, and a delicate ecological balance, the QXP supports a unique ecosystem with thousands of species that are specifically adapted to its distinct climatic and geographical conditions (Liu, Xin, et al. 2022; Liu, Xu, et al. 2022). For example, the distribution of *Ophiocordyceps sinensis*, an entomopathogenic fungus highly valued in traditional Chinese medicine, has reportedly declined on the Xizang Plateau in recent decades.

The Saxifragaceae family, a diverse group of flowering plants, constitutes a significant portion of the QXP's flora. Comprising around 10 tribes, 41 genera, and over 750 species, this family predominantly thrives in temperate zones (Folk et al. 2021). The genus *Saxifraga*, which makes up the majority of the family, is an arctic‐alpine genus with approximately 500 species, primarily concentrated in the Northern Hemisphere, particularly in the mountainous terrains of Europe and Asia, and with notable presence in the Arctic region (Gao et al. 2015; Li et al. 2023). In China, particularly within the QXP region, approximately 220 *Saxifraga* species have been documented (Gengji et al. 2018). *Saxifraga* species are valued for both their medicinal properties and ornamental appeal, owing to their unique floral traits, esthetic value, and taxonomic diversity. In traditional Chinese medicine, they are used to treat conditions such as measles, tympanitis, erysipelas, hemoptysis, piles, and hair loss (Kurosawa et al. 2024). Despite their broad distribution, there remains a significant knowledge gap regarding the environmental factors that shape their habitat preferences.

Endemic species, such as those in the Saxifragaceae family found in the QXP, play a pivotal role in maintaining ecosystem integrity, fostering biodiversity, and providing essential ecological services (Myers et al. 2000). However, climate change introduces considerable challenges, including habitat fragmentation and the growing threat of invasive species competition (Parmesan 2006). For plateau‐dwelling species, the risks are even more pronounced, given their limited populations and highly specialized evolutionary adaptations (Alexander et al. 2016; Pyšek et al. 2020). Consequently, any disturbance to their habitats could trigger a cascade of ecological disruptions, jeopardizing ecosystem health and, by extension, global biodiversity.

In light of these challenges, leveraging predictive tools such as Species Distribution Models (SDMs) has become crucial. SDMs link known species occurrences to environmental conditions and predict the probability of species occurrence under future scenarios. Among these, the MaxEnt model is widely recognized for its efficacy in ecological and biogeographical predictions, even with limited data (Phillips and Dudík 2008; Elith et al. 2006). However, it also has limitations, such as the computational intensity required due to the relationship between the number of constraint functions and sample size, which can complicate practical applications (Elith et al. 2006; Syfert et al. 2013). The primary objective of this study is to use the MaxEnt model to explore how four species within the Saxifragaceae family have been, are currently, and will likely be distributed across the QXP. We aim to unravel the complex relationships between various environmental factors—including elevation, slope, aspect, and other abiotic variables—and their influence on species distribution under different climatic conditions. By integrating climatic data and environmental determinants, this study seeks to illuminate the dynamics that govern habitat preferences and survival of these plants under past, present, and future climate scenarios.

Our study is guided by two central questions: (1) Which environmental variables predominantly shape the distribution of Saxifragaceae species in the QXP? (2) How will these distributions change under future climate conditions? Through this research, we aim to inform effective conservation strategies that will help safeguard the unique Saxifragaceae species of the QXP, ensuring their persistence amid ongoing climate change.

## 2 Materials and Methods

### 2.1 Study Area

The Qinghai‐Xizang Plateau (QXP), often referred to as the “Roof of the World” and the “Third Pole” of the Earth, is China's largest and highest plateau. It spans from the southern edge of the Himalayas in the south to the northern limits of the Kunlun, Altun, and Qilian Mountains in the north. To the west, it borders the Pamir Plateau and the Karakoram Mountains, while to the east it reaches the Qinling Mountains and the Loess Plateau in the northeast. With an average elevation exceeding 4000 m above sea level, the QXP is situated between 73°19′–104°47′E and 26°00′–39°47′N.

Covering an area of 2.5724 × 10^6^ km^2^, the QXP lies in the south‐central region of the Eurasian continent. It extends approximately 2946 km from east to west and 1532 km from south to north, accounting for 26.80% of China's total land area (Wang et al. 2020). The plateau exhibits considerable variation in elevation, with higher elevations to the west and lower elevations to the east. Its cold climate, aridity, and challenging natural conditions profoundly influence plant distribution patterns. The region's unique geography and surface characteristics give rise to a highly complex climate system (Yang et al. 2020).

The QXP experiences a climatic transition from warm‐wet conditions in the southeast to cold‐dry conditions in the northwest (Feng et al. 2020). Annual precipitation is highly seasonal and varies regionally, with the majority occurring in the summer. However, the southern portion of the plateau receives its highest precipitation in spring and autumn. In light of global warming, the QXP is undergoing substantial climatic shifts, making it a critical area for studying the impacts of climate change on alpine plant systems (Xu et al. 2016).

### 2.2 Study Species

The data for this study were collected for four representative species of *Saxifragaceae* in the alpine meadows of the Qinghai‐Xizang Plateau (QXP): *Saxifraga cernua* , *S. przewalskii* , *S. tangutica*, and *S. unguiculata* . Geographic location data for these species were obtained from six sources: (1) distribution point data collected by the research team through field investigations; (2) Global Biodiversity Information Facility (GBIF, https://www.gbif.org/); (3) China Digital Herbarium (CVH, http://www.cvh.ac.cn/); (4) National Specimen Sharing Platform (NSII, http://www.nsii.org.cn/); (5) Teaching Specimen Resource Sharing Platform (MNH, http://mnh.scu.edu.cn/); and (6) published literature on the distribution of the target species.

The collected data were processed using ArcGIS 10.8, and duplicate species distribution records were removed. For *Saxifraga cernua* , the original dataset contained 58 records. Using ENMTools, duplicates within the same grid were filtered, leaving only one point per grid for modeling. After eliminating 14 duplicates, 44 records remained for analysis. For *S. unguiculata* , 83 original records were reduced to 68 after removing 15 duplicates. In the case of *S. tangutica*, 114 records were initially available, and after eliminating 27 duplicates, 87 records were retained. For *S. przewalskii* , out of 42 original records, 12 duplicates were removed, leaving 30 records for modeling.

**FIGURE 1:**
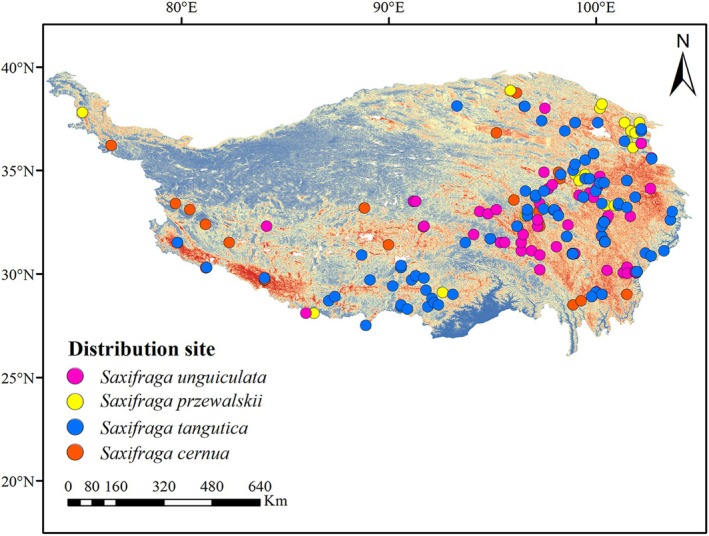
Location point map of four Saxifragaceae species.

To reduce the influence of spatial autocorrelation caused by repeated occurrences of species in the same grid, ENMTools was used to identify and remove points with high spatial autocorrelation. This process ensured that only unique geographical distribution points were retained for subsequent analysis. The final dataset was exported to Excel and converted to CSV format for use in the MaxEnt model (Figure 1).

### 2.3 Environmental Variables

Environmental variables are crucial in shaping species distributions, and the selection of appropriate variables significantly influences the accuracy of distribution models. Based on actual species distribution data and environmental variables, the probability distribution of species is inferred, allowing for the prediction of their potential distributions. To assess the impact of environmental variables on the distribution of *Saxifragaceae* species on the Qinghai‐Xizang Plateau (QXP), we followed the standard procedure outlined by Zurell et al. (2020). Initially, we identified potential environmental variables through a combination of literature review and expert consultation, considering factors such as temperature, precipitation, and elevation.

This study incorporated 19 bioclimatic and 3 topographical variables as the primary environmental factors. The bioclimatic variables were obtained from the WorldClim‐Global Climate dataset (http://www.worldclim2.0.org/) (Fick and Hijmans 2017). The dataset provides information on 19 climatic variables related to temperature and precipitation across multiple time periods: the Last Glacial Maximum (LGM), Mid‐Holocene (MH), 1970–2000, 2021–2040, 2041–2060, and 2061–2080. Each variable has a spatial resolution of 30 arc‐seconds. For the LGM and MH periods, data were derived from the Community Climate System Model version 4 (CCSM4) global climate model (GCM), while the Beijing‐based National Climate Center's BCC‐CSM2‐MR GCM was used for the future periods. These datasets form the basis of the climate scenarios. Additionally, topographical data for the QXP, including elevation, slope, and aspect, were sourced from EarthEnv (https://www.earthenv.org).

For future bioclimatic projections, this study utilized the Shared Socioeconomic Pathways (SSPs) scenarios. Over the past decade, the SSP‐Representative Concentration Pathway (RCP) framework, developed by the Intergovernmental Panel on Climate Change (IPCC), has become a key tool for climate modeling. This framework integrates radiative forcing pathways (RCPs) with five distinct SSPs. The SSP2‐4.5 scenario, which corresponds to the updated RCP4.5, was used as the primary scenario for this study. This scenario is considered the most probable development trajectory, reflecting moderate societal vulnerability and intermediate radiative forcing (Zhang et al. 2019). It aligns with current policy trends and the objectives of the Paris Agreement, avoiding extreme assumptions. In contrast, high‐emission scenarios (e.g., SSP5‐8.5) carry greater risks due to their assumption of unchecked global emissions. SSP2‐4.5 offers a more realistic baseline, considering technological advancements and policy shifts, such as the declining costs of renewable energy (Hausfather and Peters 2020). This “Middle of the Road” pathway is consistent with observed global trends, and IPCC AR6 assessments have confirmed its higher plausibility compared to low‐probability high‐emission scenarios (Rahif et al. 2023).

Characterized by moderate environmental impact and a balanced approach to mitigation and adaptation, SSP2‐4.5 is deemed the most prudent pathway for ecological studies in sensitive ecoregions like the QXP. CMIP6 evaluations have confirmed that SSP2‐4.5 represents an intermediate‐forcing scenario for the QXP, with warming trends lower than those in SSP5‐8.5 but higher than in SSP1‐2.6, consistent with “moderate pressure” thresholds (Zhang et al. 2022). Under SSP2‐4.5, temperature shifts in Qinghai Lake, for instance, are expected to be driven primarily by atmospheric warming, posing manageable threats to species like *Gymnocypris przewalskii* . In contrast, high‐emission scenarios such as SSP5‐8.5 could surpass critical thermal thresholds for these species (Dong 2023).

The potential geographic distribution of species was predicted using climate variable data under the SSP2‐4.5 scenario. All environmental variables, including both climate and topography, were resampled to a spatial resolution of 1 km using ArcGIS 10.8 and processed within the same geographical extent. To avoid collinearity and ensure the accuracy of the model, we calculated correlation coefficients among the variables. Variables with a correlation coefficient greater than 0.8 were excluded (Yan et al. 2020), leaving nine variables for the final modeling process (Table 1).

**TABLE 1:** Environmental variables selected in the MaxEnt model.

Data source	Variable category	Variable name	Abbreviation	Unit
WorldClim	Climate	Annual mean temperature	Bio1	°C
		Mean diurnal range (mean of monthly (max temp‐min temp))	Bio2	°C
		Isothermality (BIO2/BIO7) (×100)	Bio3	°C
		Temperature annual range (BIO5–BIO6)	Bio7	°C
		Precipitation seasonality (coefficient of variation)	Bio15	mm
		Precipitation of wettest quarter	Bio16	mm
EarthEnv	Topographic	Elevation	Ele	m
		Slope	Slo	°
		Aspect	Asp	°

Furthermore, we assessed the contribution of each environmental variable to the model using importance measures such as permutation importance and relative contribution. This enabled us to identify the most influential environmental variables in shaping the distribution patterns of *Saxifragaceae* species and to understand their relative significance. By adhering to this standard procedure, we ensured a rigorous and systematic approach in selecting and evaluating environmental variables for our study on the QXP.

### 2.4 MaxEnt Model Processing

The MaxEnt model was used to predict the potential distribution of *Saxifragaceae* species based on their current geographic locations and associated environmental variables. The model generates a spatial representation of habitat suitability on a scale from 0 to 1, with 0 indicating the least suitable habitat and 1 the most suitable (Shi et al. 2021).

The core principle of the MaxEnt model is to maximize entropy while satisfying specified constraints, thereby inferring the optimal probability distribution for species occurrence. It uses known distribution points and environmental variables to estimate the ecological requirements of species and simulate their potential distribution. In this study, we used MaxEnt 3.4.4 to input species data for *Saxifragaceae* and nine environmental variables. All other parameters were set to their default values, including 500 iterations, a 0.00001 convergence threshold, and a maximum of 10,000 background points (Wulff et al. 2013). The jackknife method was employed to assess the relative importance of each environmental factor (Pearson et al. 2007). For model training, 70% of the known distribution points were randomly selected, with the remaining 30% reserved for testing (Jose and Nameer 2020).

To evaluate model accuracy, we used the area under the receiver operating characteristics curve (AUC), with values ranging from 0 to 1. AUC values between 0.7 and 0.8 are classified as “fair,” between 0.8 and 0.9 as “good,” and values above 0.9 as “outstanding.” Generally, acceptable AUC values are those greater than 0.75 (Elith et al. 2006; Fourcade et al. 2014).

### 2.5 Analysis of Model Predictions

The model outputs were converted from raster to vector format using ArcGIS 10.8 and classified into four habitat suitability groups based on natural breaks (Peterson and Cohoon 1999). The Zonal Statistics tool was then employed to calculate areas of unsuitable, low‐suitability, moderate‐suitability, and highly‐suitable habitat (Brown 2014). To convert each species' continuous habitat suitability values into a binary format, a threshold of 0.1 was applied. A four‐level grading system was established to reflect the trend in species richness of *Saxifragaceae* based on suitability values, with the following categories: (0–0.1, 0.1–0.3, 0.3–0.5, and 0.5–1). Specifically, a suitability value of 0–0.1 represents unsuitable areas, 0.1–0.3 indicates low suitability areas, 0.3–0.5 corresponds to moderately suitable areas, and 0.5–1 denotes highly suitable areas.

## 3 Results

### 3.1 Model Accuracy and the Contribution of Environmental Factors

To avoid overfitting in the prediction results, we excluded environmental variables with correlation coefficients greater than 0.8 through correlation analysis. Based on model simulation results, the mean AUC values obtained from model testing using past climate data (LGM; MH), present climate data (1970–2000), and future climate scenarios (2021–2040; 2041–2060; 2061–2080) were all above 0.9. The AUC (Area Under the Receiver Operating Characteristics curve) typically ranges from 0.5 to 1.0, with higher values indicating better model performance. An AUC value closer to 1.0 suggests that the spatial distribution of the species simulated by the model closely aligns with its actual distribution. The average AUC values from model training were also above 0.9, indicating good model performance.

An internal jackknife test was conducted to assess the relative importance of various environmental factors. The results revealed that the distribution of the four *Saxifragaceae* species on the QXP was primarily influenced by topographical factors, particularly elevation (with a contribution rate exceeding 50%). Climate variables such as annual mean temperature, isothermality (Bio2/Bio7), precipitation seasonality, and precipitation during the wettest quarter, along with other factors, also played significant roles (Table 2).

**TABLE 2:** The contribution (%) of environmental variables to the MaxEnt model output of four species of Saxifragaceae.

Species name	Elevation	Slope	Bio1	Bio3	Bio15	Bio16
*Saxifraga cernua*	74.1	3.0	5.2	2.7	0.7	2.7
*Saxifraga tangutica*	60.7	0.5	1.3	8.3	5.2	10.1
*Saxifraga przewalskii*	62.8	0.8	0.1	0.0	4.7	21.9
*Saxifraga unguiculata*	57.7	1.9	0.0	1.7	18.8	5.6

Elevation was identified as the most significant factor influencing the distribution range of *Saxifragaceae* species, with its contribution exceeding 50% in the model outcomes. The environmental variables used to determine the distribution of the four *Saxifragaceae* species included slope (Slo: average of 2.02%), annual mean temperature (Bio1: average of 1.36%), isothermality (BIO2/BIO7) (Bio3: average of 2.86%), precipitation seasonality (Bio15: average of 6.12%), and precipitation during the wettest quarter (Bio16: average of 8.74%).

### 3.2 Potential Distribution of Four Species From Saxifragaceae Under Climatic Conditions at Different Periods

**FIGURE 2:**
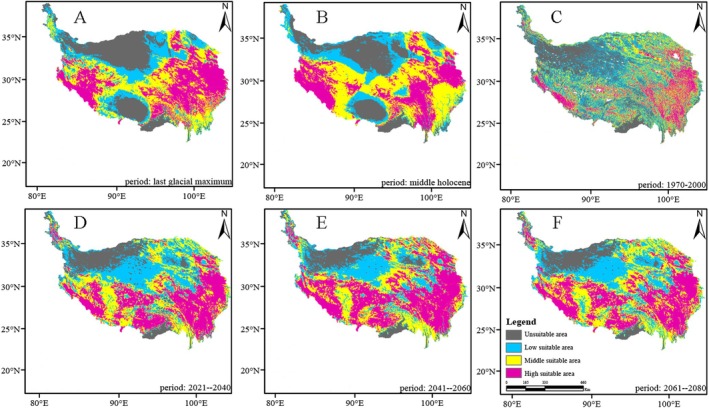
The change of distribution pattern of *Saxifraga cernua* . (A) Stand for last glacial maximum. (B) Stand for middle Holocene. (C) Stand for 1970–2000. (D) Stand for 2021–2040. (E) Stand for 2041–2060. (F) Stand for 2061–2080.

**FIGURE 3:**
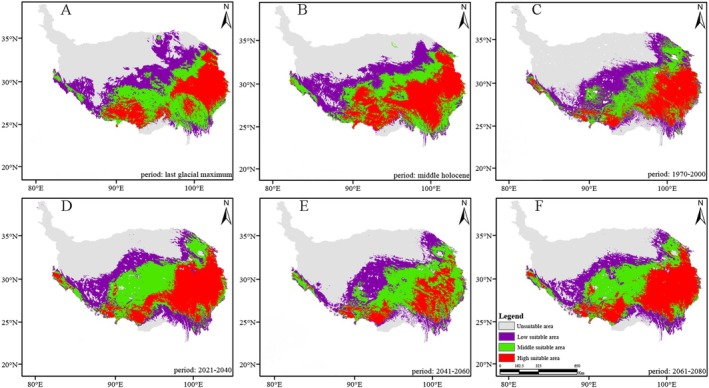
The change of distribution pattern of *Saxifraga tangutica*. (A) Stand for last glacial maximum. (B) Stand for middle Holocene. (C) Stand for 1970–2000. (D) Stand for 2021–2040. (E) Stand for 2041–2060. (F) Stand for 2061–2080.

**FIGURE 4:**
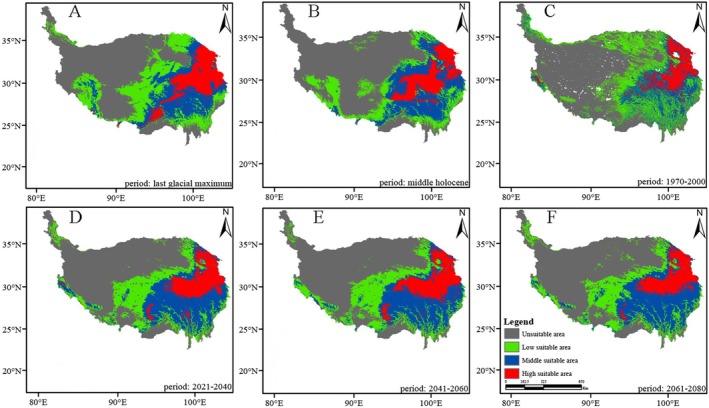
The change of distribution pattern of *Saxifraga przewalskii*. (A) Stand for last glacial maximum. (B) Stand for middle Holocene. (C) Stand for 1970–2000. (D) Stand for 2021–2040. (E) Stand for 2041–2060. (F) Stand for 2061–2080.

**FIGURE 5:**
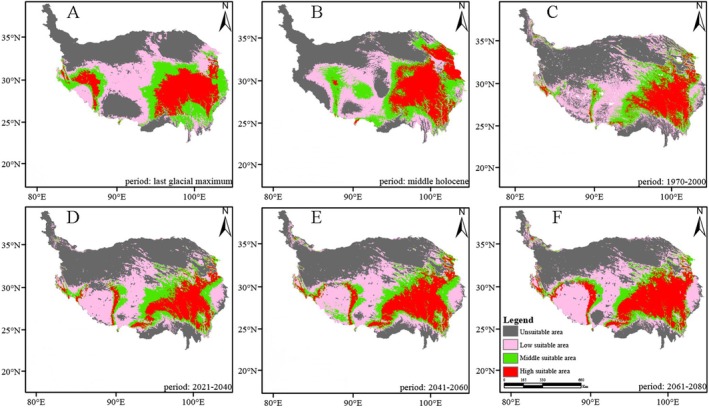
The change of distribution pattern of *Saxifraga unguiculata*. (A) Stand for last glacial maximum. (B) Stand for middle Holocene. (C) Stand for 1970–2000. (D) Stand for 2021–2040. (E) Stand for 2041–2060. (F) Stand for 2061–2080.

*Saxifraga cernua* is predominantly distributed in the southwest and southeast regions of the QXP, with the highest proportion of the high‐suitability area and an observed shift toward the southeast (Figures 2, 3, 4, 5). The suitable high regions of *S. unguiculata* and *S. tangutica* are located in the southeastern portion of the QXP. Meanwhile, *S. przewalskii* displays high suitable areas in the northeast of the plateau, with a suitable middle area located in the southeast.

As shown in Figures 2, 3, 4, 5, the area of high suitability has gradually decreased and become more fragmented from the last glacial period to the present. In contrast, from the present to the subsequent three time periods, the high‐suitability area is predicted to expand and become more concentrated within its distribution range.

### 3.3 Changes in the Potential Distribution Area

**FIGURE 6:**
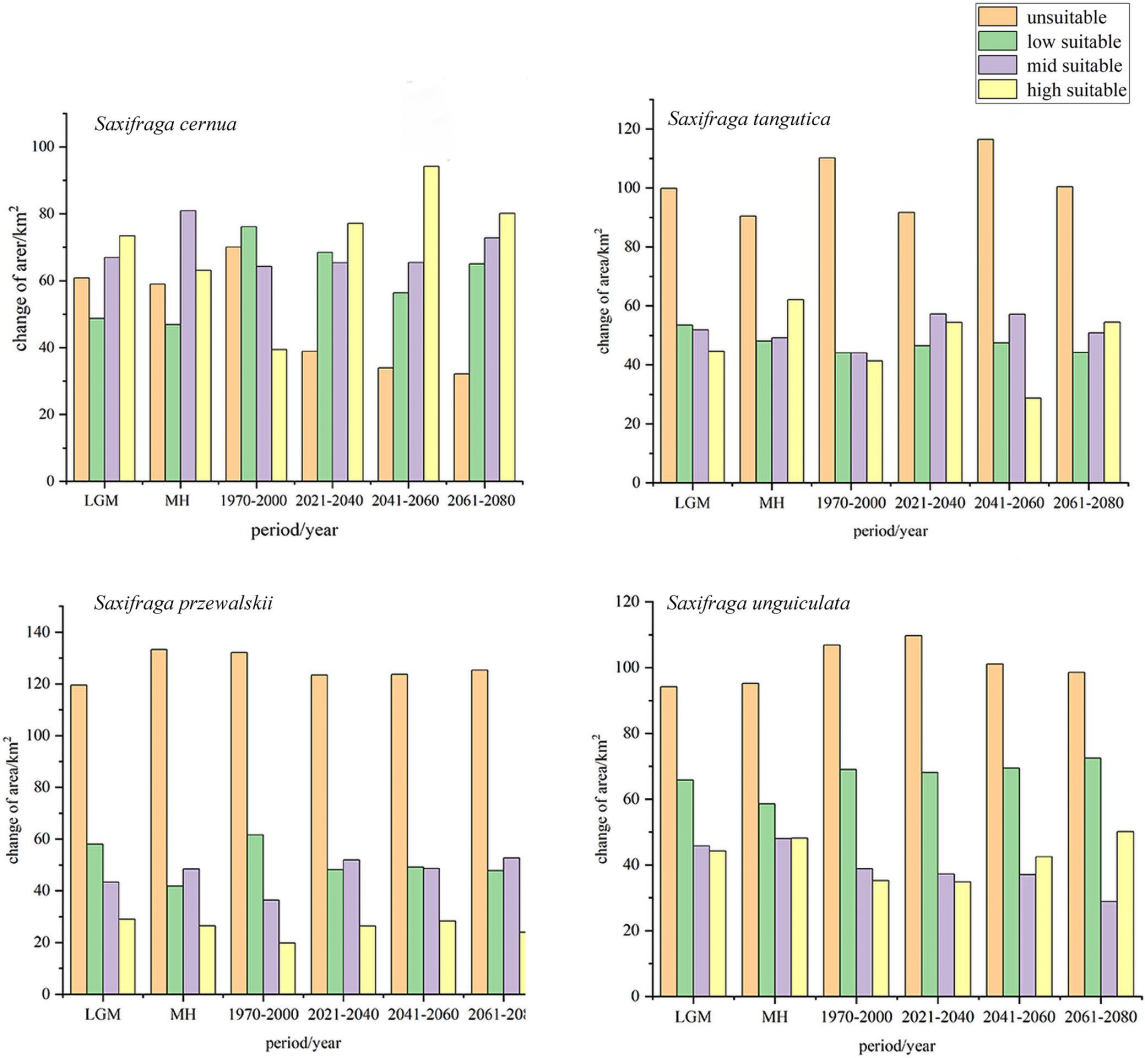
Changes of suitable area of Saxifragaceae species.

The potential distribution area of the highly suitable *Saxifragaceae* species is 1431 km^2^ (1.7% of the QXP), primarily located in the southeastern region of the plateau, as shown in Figures 2, 3, 4, 5, across past, current, and future climate scenarios. Additionally, the combined potential distribution area for the four *Saxifragaceae* species is 4740.5 km^2^ (5.2% of the QXP) under these scenarios. The potential distribution area of *Saxifragaceae* species generally decreases with increasing abundance across different climate scenarios, as shown in Table 3 and Figure 6.

**TABLE 3:** Area of the species from Saxifragaceae under climatic scenarios in different periods 10 k (km^2^).

Species	Fitness grade	LGM	MH	1970–2000	2021–2040	2041–2060	2061–2080
*Saxifraga cernua*	Unsuitable	60.86	58.99	70.09	38.98	33.90	32.10
	Low suitable	48.72	46.94	76.20	68.48	56.42	65.02
	Mid suitable	66.96	80.94	64.26	65.40	65.47	72.75
	High suitable	73.46	63.13	39.45	77.14	94.22	80.13
*Saxifraga tangutica*	Unsuitable	99.87	90.55	110.26	91.72	116.51	100.36
	Low suitable	53.52	48.19	44.10	46.55	47.53	44.26
	Mid suitable	51.96	49.20	44.10	57.32	57.19	50.88
	High suitable	44.65	62.05	41.37	54.41	28.77	54.49
*Saxifraga przewalskii*	Unsuitable	119.54	133.24	132.11	123.43	123.75	125.36
	Low suitable	58.04	41.77	61.62	48.20	49.23	47.90
	Mid suitable	43.36	48.48	36.46	51.93	48.66	52.67
	High suitable	29.06	26.52	19.81	26.44	28.37	24.07
*Saxifraga unguiculata*	Unsuitable	94.22	95.26	106.89	109.80	101.06	98.59
	Low suitable	65.78	58.60	68.98	68.12	69.36	72.50
	Mid suitable	45.75	47.99	38.86	37.25	37.06	28.83
	High suitable	44.24	48.15	35.27	34.84	42.53	50.08

Abbreviations: LGM, Last Glacial Maximum; MH, Middle Holocene.

Over time, unsuitable areas for the four *Saxifragaceae* species have steadily decreased, while highly suitable regions have expanded. Among the species, the unsuitable areas for *S. tangutica*, *S. przewalskii* , and *S. unguiculata* accounted for 50% of the total area of the QXP under various climatic scenarios.

During the Last Glacial Maximum, the largest suitable habitat area was for *Saxifraga cernua* (73.46 km^2^, accounting for 29.4% of the QXP), while the smallest was for *S. tangutica* (29.06 km^2^, accounting for 11.6% of the QXP). In the Middle Holocene, the largest suitable area shifted to *S. przewalskii* , and during the 1970–2000 period, it was for *S. tangutica*. In the subsequent periods (2021–2040, 2041–2060, and 2061–2080), *S. cernua* is projected to have the largest high‐suitability area, as shown in Table 3.

In comparison to the medium and high‐suitability areas, the low‐suitability areas represent a smaller proportion for the four *Saxifragaceae* species. Figures 2, 3, 4, 5 illustrate that these low‐suitability areas are primarily concentrated in the northwestern part of the QXP. In contrast, the medium‐ and high‐suitability areas occupy a larger proportion, with high‐suitability areas covering over 6.0 × 10^5^ km^2^. Across the four species, the unsuitable areas have decreased in size over time, indicating an overall contraction. Conversely, highly suitable areas generally expanded from the past to the future, except for *S. przewalskii* .

### 3.4 Migration Trend of Four Species From Saxifragaceae With Elevation Gradient at Different Periods

The mean elevation of the potential distribution of *Saxifragaceae* species in the specified area decreased as elevation increased, as shown in Tables 3 and 4. This suggests that the distribution range of *Saxifragaceae* species generally contracted at higher elevations. In the highly suitable areas, the average elevation of the potential distribution for *Saxifragaceae* species was 3332 m across all periods (Tables 3 and 4).

**TABLE 4:** The average elevation of land that could potentially support life for four different species under the present climate conditions.

Species	Low	Mid	High	Unit
*Saxifraga cernua*	4100	3900	3800	m
*Saxifraga tangutica*	3800	3650	3400	m
*Saxifraga przewalskii*	3200	3000	2260	m
*Saxifraga unguiculata*	4200	3952	3500	m

Among the four *Saxifragaceae* species, *Saxifraga cernua* was observed at the highest average elevation, while *S. przewalskii* was found at the lowest average elevation. Generally, areas with unsuitable or low suitability for these species were concentrated at higher elevations, while those with medium to high suitability were located at lower elevations. The areas of high suitability tended to shift toward lower elevations, primarily toward the southeast of the QXP, as well as northern Yunnan and western Sichuan. Additionally, regions with medium and high suitability were becoming increasingly concentrated. According to Tables 3 and 4 and Figures 2, 3, 4, 5, these middle and highly suitable areas are expected to continue migrating southward under projected climate change.

## 4 Discussion

### 4.1 Influences of Environmental Factors on the Potential Distribution of the Species From Saxifragaceae

Environmental variables consist of both biological and abiotic components, with climatic factors playing a central role in determining a species' distribution and dispersion across large areas. Species distribution is also influenced by broad‐scale hydrothermal conditions, such as temperature and precipitation. Biological and environmental factors, which have a more localized impact, can interact in complex ways to affect distribution. However, at larger geographical scales, these interactions may become less significant due to reduced interspecies interactions (Thuiller et al. 2005). Accordingly, our study employed common large‐scale bioclimatic factors to simulate the distribution range and patterns of four *Saxifragaceae* species (Hijmans et al. 2005). Despite existing studies on the topic, gaps remain in understanding the species distribution of *Saxifragaceae*. To address this, our findings highlight that elevation, slope, annual mean temperature (Bio1), isothermality (Bio3), precipitation seasonality (Bio15), and precipitation during the wettest quarter (Bio16) are key environmental variables influencing the potential distribution of *Saxifragaceae*, with elevation playing the most significant role.

Our results underscore the prominent role of elevation in shaping species distributions, offering new insights into species‐environment interactions, particularly in the context of climate change. Previous research has documented upslope shifts in plant species in response to climate warming in the European Alps (Lenoir et al. 2008). Interestingly, our results indicate that *Saxifragaceae* species on the QXP exhibit a trend of shifting their potential distribution to lower elevations. This observation aligns with findings from other studies (Rumpf et al. 2018), which reported that, under more optimistic climate scenarios, *Saxifraga* species in the Himalayas are projected to shift their ranges to lower elevations, likely due to water availability rather than temperature changes (Qiu et al. 2024). A similar pattern was observed in studies comparing historical and current plant distributions in California (Crimmins et al. 2011). Additionally, research in Gongga Mountain, the highest peak in the HDM, found that a notable percentage of plant species showed significant downslope shifts (Zu et al. 2021).

Additional research supports these findings, with notable elevation‐related changes observed in various terrestrial taxa. For instance, aroids showed a marked decline with height, whereas orchids and piperoids increased, and ferns exhibited a hump‐shaped pattern, with the highest richness at mid‐altitudes (Krömer et al. 2013). Studies on plant diversity and productivity along altitude gradients in the northern QXP reveal that at four different altitudes (4485, 4535, 4585, and 4635 m), sedges have shifted from relative dominance to absolute dominance, while the importance of forage species has decreased at different rates. As altitude increases, legumes become more important, but their significance diminishes at higher elevations. Biodiversity indices, such as species richness, Simpson's index, Shannon‐Wiener index, and Pielou's evenness index, were significantly higher at mid‐elevations, indicating a hump‐shaped relationship with elevation. Biomass showed a positive correlation with both Shannon‐Wiener and Simpson indices (*p* < 0.05), but not with Pielou's evenness index or functional group importance (*p* > 0.05). These findings suggest that productivity and diversity in the alpine meadows of northern Xizang are strongly influenced by elevation (Li et al. 2017).

The Tibetan Plateau's higher elevations and drier conditions create unique challenges under climate change. Research has shown that warming rates are faster at higher altitudes, and weather station data from elevated regions suggest reduced snowfall and rainfall despite an overall increase in precipitation across the region. This combination may contribute to reduced humidity, which, coupled with exposure to strong winds and intense solar radiation, is putting high‐altitude plants under increasing moisture stress. This phenomenon also correlates with the higher contributions of environmental factors such as Bio15 and Bio16 to the fitness zone. Moreover, forests of Qinghai spruce (*Picea crassifolia*), a conifer species endemic to China and a dominant treeline species on the northern Tibetan Plateau, are either dying off or shifting to lower altitudes (Qiu 2015).

Species distribution is fundamentally influenced by environmental conditions, particularly climate variables such as temperature and precipitation. These factors affect key biological processes, such as dispersal ability, home range size, and survival under unfavorable conditions (Sharma and Raghubanshi 2006). Climate‐based factors can synchronize critical biological processes, including dispersal ability, home range size, and survival. However, the impact of climate variables is not universal, as other edaphic and topographic factors, along with interactions between biotic and abiotic environments, can also significantly affect species distribution (Norberg et al. 2019).

Our study emphasizes the importance of environmental variables, particularly elevation, in shaping the potential distribution of *Saxifragaceae* species. Elevation serves as a proxy for numerous ecological gradients in mountainous regions like the QXP, encompassing not only climate but also soil composition, vegetation types, and biological interactions. By integrating large‐scale bioclimatic factors with more localized, location‐specific variables such as elevation, this approach not only substantiates the effects of broad‐scale hydrothermal conditions but also highlights the complexity of species‐environment interactions at finer scales. Further studies will be essential to unravel the intricate interplay between these environmental factors and their collective impact on species distribution.

### 4.2 Species Potential Distribution of the Family Saxifragaceae

In this study, the MaxEnt model was used to analyze how the distribution areas of four *Saxifragaceae* species on the QXP changed over time across different periods. The results demonstrate that we used the Area Under the Curve (AUC) value to evaluate the predictive performance of the model, a commonly used criterion in ecological studies to assess the accuracy of niche modeling and species distribution modeling (Allouche et al. 2006). AUC is also extensively utilized to evaluate the accuracy of habitat suitability models (Allouche et al. 2006; Luis et al. 2017). The average AUC values across all periods in this study were greater than 0.9, meeting the standard for reliable modeling. Therefore, the predicted results are considered to be both accurate and dependable.

We conducted a thorough investigation to explore the distribution of four *Saxifragaceae* species on the QXP under various climatic scenarios (past, present, and future). Our findings indicated that the species' distribution range was largest in the southeastern QXP, particularly in highly suitable regions. Climate change emerged as a significant factor influencing the potential distribution of *Saxifragaceae* species on the QXP. Specifically, annual precipitation and mean temperature during the driest quarter had substantial impacts on their distribution, likely linked to the elevation gradients, which in turn influence key climate factors such as humidity and temperature (Tsiftsis et al. 2008).

Our analysis suggests that the distribution of *Saxifragaceae* species is primarily climate‐driven, as the total gain in the MaxEnt model was largely influenced by temperature and precipitation. Across past, present, and future climate scenarios, the distribution of *Saxifragaceae* species was observed to decrease with increasing elevation. This trend can be explained by the fact that higher elevations typically experience lower temperatures and higher precipitation, which alter the microclimate and can negatively impact plant growth and survival (Moradi and Oldeland 2019). Species from *Saxifragaceae* are adapted to specific environmental conditions, and fluctuations in temperature and precipitation can disrupt their ability to survive and reproduce. During periods of global cooling, past climate scenarios have shown that many plant species shifted their ranges to lower elevations (Lomolino et al. 2010; Sanders and Rahbek 2012; Liu, Xin, et al. 2022; Liu, Xu, et al. 2022). Similarly, in present‐day climate scenarios, species from *Saxifragaceae* have been observed shifting their distributions to lower elevations in response to rising temperatures and changing precipitation patterns. Future climate projections predict continued warming and more erratic precipitation, which could drive further shifts in species ranges. If the rate of climate change is too rapid, some species may struggle to adapt quickly enough and could face extinction.

Our investigation focused on a gradient that extended well above mid‐elevation, yet the observed decline in species diversity at higher elevations may also be linked to the conical structure of mountains, which reduces the available habitat area (Liu, Xin, et al. 2022; Liu, Xu, et al. 2022). Previous studies have demonstrated that species richness is highest in mid‐elevation zones and decreases in both the high and low elevation extremes. Our findings align with this, as we observed peak distributions of *Saxifragaceae* species at middle elevations. Additionally, the higher distribution of *Saxifraga przewalskii* at lower elevations may be related to the higher temperatures in that region, which are more suitable for its growth.

### 4.3 Potential Distribution and Migration Trends of the Species From Saxifragaceae in Different Climate Periods

The observed habitat suitability trends among *Saxifraga* species highlight the diverse adaptive strategies plants use in response to environmental shifts. *Saxifraga cernua* exhibits impressive evolutionary adaptability, potentially attributed to genetic factors, phenotypic plasticity, and possible symbiotic relationships (Birks and Willis 2008). In contrast, the consistent presence of *Saxifraga unguiculata* across varied habitats suggests a broader ecological tolerance, indicating its ability to endure diverse environmental conditions (Holt 2009). However, the fact that *Saxifraga przewalskii* predominantly inhabits unsuitable habitats raises significant conservation concerns. This highlights the need for measures such as habitat restoration or assisted migration to safeguard the species (Pellissier et al. 2013). Continued monitoring and research are crucial to gain a more comprehensive understanding of the species' ecological dynamics in the face of ongoing environmental changes (Vogt et al. 2013).

In the current climate scenario, the four species primarily occupy the intersection zone between the southeastern QXP, western Sichuan, and northern Yunnan. High elevation, cold temperatures, and aridity are the main environmental factors influencing these suitable habitat areas (Cheng and Wu 2007). Under climate change scenarios, from the Last Glacial Maximum to the Middle Holocene, the highly suitable habitats for these four species shifted eastward, from the northwest to the southeast of the QXP. These areas were predominantly located in the southeastern QXP, western Sichuan, and northern Yunnan.

In future climate scenarios, the high and medium‐suitability habitat areas for these four species are expected to increase slightly compared to the present climate. This shift may be attributed to the availability of moisture and low temperatures in these regions, which will continue to support viable distributions as the climate changes (Chen et al. 2011; Fei et al. 2017). However, the area and distribution patterns of potentially suitable habitats are likely to remain relatively stable. This stability could be due to the complex topographic structure of the Qinling‐Qilian Shan‐Kunlun Mountain system along the eastern margin of the QXP, which limits the long‐distance dispersal of these species. Additionally, the continuous distribution of valleys and mountain ranges in lake basins offers secure local refuges, creating a unique, stable, and narrow distribution pattern. As a result, future climate change is unlikely to significantly threaten these species.

### 4.4 Limitations and Conservation Implications

Effectively allocating conservation resources requires identifying areas of high conservation value. Under extreme climatic conditions, areas where species can find refuge and persist are known as biological sanctuaries. These sanctuaries are crucial for safeguarding the survival of organisms and biodiversity under unfavorable conditions, often marked by significant contraction in species' distribution ranges (Tzedakis et al. 2002). Through a comparative analysis of *Saxifragaceae* species' habitats across past, present, and future scenarios, we have determined that the southeastern region of the QXP represents the most favorable habitat for these species. This conclusion is based on several factors: first, the relatively low elevation of approximately 3000 m; second, the temperate climate influenced by the Brahmaputra Valley, which allows for the ingress of summer monsoons; and third, the abundant precipitation in this region. As a result, we identify the southeastern QXP as a sanctuary and key distribution zone for *Saxifragaceae* species in the face of future climate change.

In light of these findings, we propose the establishment of a nature reserve in the southeastern QXP to protect *Saxifragaceae* species. According to our results, the overall suitable distribution area for the four species across the entire QXP is very limited, with high‐suitability areas concentrated only in the southeast. The potential distribution area of highly suitable habitat for *Saxifragaceae* species covers 1431 km^2^ (1.7% of the QXP), and this area is expected to shift southward. In over 80% of the QXP, *Saxifragaceae* species are scarcely distributed. Therefore, the creation of a protected area in the southeastern region of the QXP is essential. This reserve would serve as a model for climate change adaptation, supporting dynamic conservation strategies and reducing the risks of endangerment and extinction due to climate change.

However, due to inherent limitations in integrating all influencing factors within species distribution models (SDMs), our predictions represent idealized habitats under simplified conditions. This study focuses specifically on climatic and topographic drivers of species distribution, using the SSP2‐45 medium‐emission pathway as it reflects the most likely future scenario in alignment with current policy trends. Nonetheless, this approach does not account for the differential impacts of alternative emission scenarios on *S. cernua* , *S. tangutica*, *S. przewalskii* , and *S. unguiculata* across the QXP. Furthermore, our model assumes that species distributions are primarily shaped by climate and topography, with unrestricted colonization of suitable habitats. Critical constraints such as biotic interactions (e.g., evolutionary history, dispersal capacity, and species competition), human activities (e.g., land‐use change), and abiotic factors (e.g., soil properties and microhabitat heterogeneity) have not been incorporated. Future research should incorporate these multidimensional factors into more comprehensive modeling frameworks to improve prediction accuracy and provide a clearer understanding of the spatial distribution patterns of these *Saxifragaceae* species under changing climate conditions.

## 5 Conclusion

Climate change has a significant impact on the distribution of endangered *Saxifragaceae* species on the Qinghai‐Xizang Plateau (QXP). Our study highlights the importance of integrating climate change projections into species distribution models to better understand potential impacts and inform effective conservation strategies. It also underscores the need for adaptive management approaches that account for the dynamic nature of species distributions in response to shifting climatic conditions. By analyzing data from historical, current, and future climate scenarios, we identified several key environmental variables that influence the potential distribution of endangered *Saxifragaceae* species on the QXP, including elevation, slope, mean annual temperature, isothermality, precipitation seasonality, and precipitation during the wettest quarter. Our findings show that the mean elevation of suitable habitats for these species ranges from 2260 to 4150 m under different climate scenarios. Notably, the southeastern QXP emerged as the most suitable region for *Saxifragaceae* species, both under current conditions and future projections, with a general trend of decreasing distribution with increasing elevation. To ensure the long‐term survival of these endangered species, we recommend prioritizing the establishment of protected areas in the southeastern QXP, as well as in western Sichuan and northern Yunnan. This targeted conservation approach will help safeguard *Saxifragaceae* species amidst ongoing climate change and other environmental challenges.

## Author Contributions

**Yang Lv:** visualization (equal), writing – original draft (lead). **Zhaxi Cairang:** writing – original draft (equal). **Chenglin Sun:** visualization (equal), writing – original draft (equal). **Xu Su:** conceptualization (lead), funding acquisition (lead), writing – review and editing (equal). **Yuping Liu:** conceptualization (equal), writing – review and editing (equal). **Yonghui Zhou:** writing – original draft (supporting). **Kaiyue Wei:** data curation (equal). **Xu Feng:** formal analysis (equal). **Jieqiong Lei:** writing – original draft (supporting). **Yinghui Zheng:** writing – original draft (supporting). **Xuanlin Gao:** writing – original draft (supporting). **Mir Muhammad Nizamani:** writing – review and editing (equal).

## Disclosure

Publisher's note: All claims expressed in this article are solely those of the authors and do not necessarily represent those of their affiliated organizations or those of the publisher, the editors, and the reviewers. Any product that may be evaluated in this article, or claim that may be made by its manufacturer, is not guaranteed or endorsed by the publisher.

## Conflicts of Interest

The authors declare no conflicts of interest.

## Data Availability

The distribution point data sets analyzed in this study are available in the Global Biodiversity Information Facility (GBIF: https://www.gbif.org/), Chinese Virtual Herbarium (https://www.cvh.ac.cn/), National Specimen Sharing Platform (NSII, http://www.nsii.org.cn/) and Teaching Specimen Resource Sharing Platform (MNH, http://mnh.scu.edu.cn/). The environmental variables were obtained from the WorldClim‐Global Climate dataset (http://www.worldclim2.0.org/) and EarthEnv (https://www.earthenv.org).
